# A qualitative study on the impact of COVID-19 on the behavior and attitudes of smokers and non-smokers in South Korea

**DOI:** 10.1186/s12889-021-12079-8

**Published:** 2021-11-01

**Authors:** Jieun Hwang, Hae-ryoung Chun, Eunsil Cheon

**Affiliations:** 1 College of Health Science, Dankook University, 119, Dandae-ro, Dongnam-gu, Cheonan-si, Chungnam, 31116 Republic of Korea; 2 Graduate School of Public Health, Seoul National University, 1 Gwanak-ro, Gwanak-gu, Seoul, 08826 Republic of Korea

**Keywords:** Smoking, COVID-19, Attitudes, Tobacco products, Korea

## Abstract

**Background:**

The COVID-19 pandemic has affected all aspects of human society, including education, culture, and the economy, and has also introduced changes in people’s health behaviors such as drinking alcohol, nutrition intake, and practicing healthy living. This study conducted qualitative research in the Korean context to examine the changes in the smoking behavior of smokers and secondhand smoke exposure of non-smokers during the COVID-19 pandemic.

**Methods:**

Focus group interviews were conducted with 36 Korean participants (18 men and 18 women). The groups were composed of cigarette smokers, e-cigarette users, heated tobacco product users, and non-smokers.

**Results:**

During the pandemic, it was found that there was an increase in the frequency of use, irrespective of the tobacco product, in users who refrained from social interaction and worked or studied from home. Users who continued to be socially active increased the amount used with each usage. Smokers showed a tendency to avoid smoking rooms and to smoke alone in places unoccupied by people. In addition, non-smokers’ exposure to secondhand smoke did not decrease, but since non-smokers used masks, they reported more relief from the risk of exposure to secondhand smoke than before.

**Conclusions:**

Despite smokers being a high-risk group for COVID-19, the risk did not result in smoking cessation among smokers. Therefore, policies and educational campaigns to raise awareness about the dangers of smoking and to encourage smoking cessation are needed in the future.

## Background

Coronavirus disease 2019 (COVID-19), which originated in China, began to spread to other countries from January 2020 onwards, spreading rapidly to most countries, excluding only a few [1]. Accordingly, the World Health Organization declared a public health emergency of international concern on January 31, 2020 and subsequently a pandemic on March 11, 2020. As of March 17, 2021, there were 121,464,666 confirmed cases and 2,684,093 deaths worldwide [2].

The COVID-19 pandemic has resulted in the isolation of nations and people, and brought about profound changes in societies, economies, and cultures worldwide. The pandemic has negatively affected the education system, with the shutting down of schools and the move to online classes; the tourism industry; and face-to-face service industries, such as restaurants and hotels [3, 4]. Some industries, such as the video conferencing service industry and delivery services have benefited [5, 6].

The pandemic has also affected health behaviors. Since the outbreak, alcohol intake patterns have both increased and decreased, physical activity has reduced due to more sedentary lifestyles, and food consumption and eating patterns have become unhealthy [7, 8]. Furthermore, the pandemic has led to an increase in mental health problems such as stress, anxiety, depressive symptoms, insomnia, denial, anger, and fear [9].

Smoking behaviors have also changed. Both the initiation and the amount of smoking have increased. According to Cancello’s study in Italy, after the lockdown, 38% of habitual smokers reported an increase in tobacco consumption [10]. In Ren’s study in China, 30.1% of smokers increased their cigarette use, and in Chen’s study, 25% of smokers smoked more [11, 12]. Đogaš’s study in Croatia reported that the number of cigarettes used increased significantly [13]. Smokers who had given up smoking, thought about smoking or restarted smoking during the pandemic [14]. However, in some studies, an increase in the intent to quit smoking, an attempt to quit smoking, or a decrease in the amount of smoking were also reported [14].

It was reported that these behavioral changes were caused by boredom, the lack of social access, and disruption of daily life during the pandemic [15]. As the pandemic induced stress, the amount of smoking increased to relieve that stress [16]. Consequently, changes in smoking behavior can be attributed to the psychological changes experienced by smokers during the COVID-19 pandemic [12].

However, very few studies have examined the relationship between the psychological factors caused by the pandemic and changes in smoking behavior. Studies have examined the stress, anxiety, and depression of smokers after the pandemic, but have not delved into how these psychological factors work and affect the smoking behavior of smokers. Additionally, as with most quantitative studies, interpretations of the reasons for changes in smoking behavior were limited. In particular, it has not been determined whether exposure to secondhand smoke changed for a majority of non-smokers and whether these factors changed their perceptions or attitudes toward existing smoking or secondhand smoke.

Therefore, this study conducted qualitative research to examine changes in the smoking behavior of smokers during the COVID-19 pandemic. Moreover, it explored whether changes in exposure to secondhand smoke were experienced by non-smokers and whether their attitudes toward smoking and secondhand smoke changed. To this end, this study conducted focus group interviews (FGIs) as a qualitative research method to understand and interpret the behavioral and attitudinal changes of the study subjects. Based on the study findings, we propose an effective tobacco control policy for the pandemic, and suggest a direction of research for promoting policies based on scientific research.

Since the first confirmed cases of COVID-19 on January 20, 2020, South Korea has experienced three waves of COVID-19 [17]. South Korea ranked second in the world for the number of confirmed cases, with approximately 5000 confirmed cases 43 days after the first COVID-19 patient. Nonetheless, through the efforts of the Ministry of Health and Welfare, the Korea Disease Control and Prevention Agency, and advanced quarantine systems, it succeeded in reducing the number of new cases [17]. This study makes an academic contribution to the existing body of literature as it shares interview data from South Koreans who have experienced such a dynamic pandemic, and attempts to contribute to promoting effective policies.

## Methods

### Sample and recruitment

FGIs were conducted for this study. Through a survey agency, a total of 36 Korean participants were recruited and divided into six groups according to gender, age, and smoking status: adult tobacco user men, adult tobacco user women, adult tobacco non-user men and women, tobacco user boys, tobacco user girls, and tobacco non-user boys and girls. To observe changes in attitudes, and changes according to age and gender, a group of adolescents aged 16–19 was enlisted, and an adult group aged 20–49 was signed up. The adult group was limited to those who had been working for more than a year because the changes in lifestyle would then be more conspicuous, such as commuting to and from work due to COVID-19. At the time of recruitment, the participants provided information about their current smoking status and the types of tobacco products they used. Current smokers were limited to those who had used each type of tobacco product for 6 months or more. Tobacco user groups were classified according to products used: cigarettes, heated tobacco products (HTPs), and e-cigarettes (including the closed system vaporizer; CSV); and both single- and dual-product users were included. Those who engaged in tobacco manufacturing, distribution, sales, the medical field, and media/advertising/marketing businesses were excluded.

### Interview protocol and data collection

Interviews were conducted by a male moderator from November 16–21, 2020 based on a semi-structured questionnaire. The questionnaire, developed by the three authors, was completed after being reviewed by an external expert and was customized for adult smokers, adult non-smokers, adolescent smokers, and adolescent non-smokers. It included the following items: the participants’ current and past smoking behaviors, smoking perceptions, subjective health status, and changes during the pandemic. Interviews were conducted for about an hour in the FGI room of the survey agency’s office in Seoul, Korea, with six people per group. With the consent of the participants, the interviews were recorded, and after the interview, the interview content was transcribed. In the case of students, parental consent was obtained before participation in the interviews. All participants received 70,000 won (about 62 US$) in cash. Ethical issues in the study were reviewed and approved by the Institutional Review Board of the Seoul National University (IRB No. 2010/001–018).

### Data analysis

The first author coded the six transcripts using MAXQDA software. Coding was performed using a line-by-line open coding approach. The second author repeated the coding using the same method. The two codes were compared and refined to create categories, and the final code was completed. The relevant transcript extracts in the final code were finalized through discussion and agreement between the three authors.

## Result

### Participant characteristics

The total number of participants was 36 (18 men and 18 women) (Table 1). There were 18 teenagers (9 male and 9 female students), with one high-school freshman student, four second-grade students, and 13 third-grade students. A total of 15 adult office workers who were in their 20s or older (9 males and 6 females) were included, with an average age of 33.4 years. Of the 36 participants, 25 were smokers and 11 were non-smokers. In terms of tobacco product: 13 were using cigarettes, 5 were using e-cigarettes, and 7 were using HTPs. Nine participants were dual or multiple tobacco product users.

**Table 1:** Participants’ characteristics

Group	No.	Age	Sex	Type of tobacco products
Group 1	1	33	Male	Cigarette
	2	30	Male	Cigarette
	3	27	Male	Heated tobacco product
	4	43	Male	Heated tobacco product /Cigarette
	5	32	Male	e-cigarette/Cigarette
	6	43	Male	Heated tobacco product /Cigarette
Group 2	7	36	Female	Heated tobacco product /Cigarette
	8	44	Female	Heated tobacco product /Cigarette
	9	29	Female	Cigarette
	10	27	Female	Cigarette
	11	38	Female	e-cigarette/Cigarette
	12	33	Female	e-cigarette (CSV)
Group 3	13	33	Female	–
	14	32	Female	–
	15	47	Female	–
	16	44	Male	–
	17	29	Male	–
	18	22	Male	–
Group 4	19	19	Male	e-cigarette (CSV)
	20	19	Male	Cigarette/ e-cigarette
	21	19	Male	e-cigarette (CSV)
	22	18	Male	Heated tobacco product /Cigarette
	23	18	Male	Cigarette
	24	18	Male	Cigarette
Group 5	25	19	Female	Cigarette
	26	19	Female	Heated tobacco product /Cigarette
	27	19	Female	Cigarette
	28	19	Female	Cigarette/ Heated tobacco product
	29	19	Female	Cigarette
	30	18	Female	Cigarette
	31	19	Female	Cigarette
Group 6	32	19	Female	–
	33	19	Female	–
	34	19	Male	–
	35	17	Male	–
	36	19	Male	–

### Themes and categories

The classification of themes and categories that emerged from the interviews is shown in Table 2. Themes were divided into five categories: changes in daily life; changes in smoking behavior (smoking place, smoking amount and frequency, products used); changes in smoking perception; subjective mental health; and exposure to secondhand smoke.

**Table 2:** Themes and categories

Themes	Categories
Changes in daily life	Changes in daily lives among students
	Changes in daily lives among adults
	Changes in daily lives for both students and adults
Changes in smoking behavior	No change
	Smoking place
	Smoking amount and frequency
	Tobacco product
Changes in smoking perception	Smoking room
	Smoking
Changes in subjective mental health	Stress
Change in exposure to secondhand smoke	Continuous exposure to secondhand smoke

### Changes in daily life

COVID-19 changed the daily lives of both students and adults. Most schools were shut, but classes were taught online. Group activities such as drama circle were stopped, and leisure activities were restricted due to the closure of facilities like study cafes and PC rooms. Among adults, some were isolated due to the shutting down of their businesses, and there were cases where the form of work changed due to telecommuting. Like the changes experienced by students, social and outdoor activities such as social gatherings and sports almost completely disappeared.

*“During the pandemic, there were days when I used to go to school, but now I rarely go.” (M 18, HTP and cigarette user)*

*“I worked at the airport, but now I am resting because the company is closed after COVID-19.” (W 27, cigarette user)*

*“I had been in business for about 13 years; due to the pandemic, it closed on the 15th of May.” (W 38, e-cigarette and cigarette user)*

*“Gatherings with friends has decreased a lot.” (W 33, non-smoker)*

### Changes in smoking behavior

#### No change

Most smokers responded that there was no change in their smoking behaviors after the COVID-19 pandemic. They recognized that their behavior had not changed since there had been no smoking cessation or discontinuation of tobacco products.

*“Nothing has changed in particular about smoking.” (M 18, cigarette user)*

*“There is no change. The smoking habit is similar, and it all just seems the same. Only wearing masks. That is all.” (F 19, cigarette user)*

*“It does not seem like much.” (M 33, cigarette user)*

*“Personally, I do not notice any change.” (F 33, e-cigarette user)*

#### Changes in smoking place

On the other hand, there were changes in smokers’ smoking places, smoking frequency, smoking amount, and types of tobacco products since COVID-19. These changes were observed in respondents who previously said there is no change.

After COVID-19, smokers’ attitudes toward smoking places became very different. Smokers avoided smoking areas and smoking rooms where many people gathered and began smoking alone to avoid people. Some companies recommended not to smoke in groups and to refrain from talking with others while smoking. Additionally, spacing policies between smoking spots were implemented by some companies.

*“I do not go to places with a lot of people; instead, I find a corner.” (W 38, e-cigarette and cigarette user)*

Existing smoking rooms and smoking areas were avoided due to the psychological factor of being bothered by smoking with others in an enclosed space, and feeling anxiety and fear. Additionally, those who had confirmed cases around them tended to be more careful so as not to be infected, resulting in the change in smoking place.

*“When there is no one, I smoke quickly and come out. That is what I care about after COVID-19.” (M 33, cigarette user)*

These changes in places showed different patterns depending on the tobacco product used. HTP or e-cigarette users smoked regardless of place compared to cigarette smokers. However, cigarette smokers mostly smoked outdoors with no people around, and they did not complain of any discomfort about this, despite the change. Some users tended to select products according to the smoking place, but mainly used HTPs or e-cigarettes indoors, and cigarettes outdoors.

*“I wanted to smoke at home during the pandemic, so I started using e-cigarettes.” (M 19, cigarette and e-cigarette user)*

Considerable rejection and anxiety were also shown regardless of the type of tobacco product. This was explained by reluctance, anxiety, and fear about the characteristics of the smoking room, such as being confining, densely crowded, with people having to take off their masks.

*“I never go into a box-shaped smoking area. Box type booths are a little worrying.” (W 36, HTP and cigarette user)*

#### Changes in smoking amount and frequency

Changes in work and study environments due to COVID-19 affected changes in smoking frequency and smoking amount. In case of working from home or taking online classes, there was often no observer, and it was possible to go out frequently. However, in case of office workers, due to unsuitable smoking places or time constraints, the amount of smoking increased because people tended to smoke a lot at once. There also were opinions that smoking frequency decreased after going to work due to the presence of co-workers; however, the overall smoking amount or smoking frequency did not change, or increased after the pandemic.

*“I do not go to work, so my smoking has increased. I smoke anytime at home, right on the roof.” (W 27, cigarette user)*

*“We are taking online classes and just smoke freely. Because my parents are at work. I do not have to worry about being scolded by the teacher, so I think the frequency is increasing.” (W 19, cigarette user)*

*“I smoke three or four cigarettes.” (W 44, HTP and cigarette user)*

#### Change in tobacco product

Most smokers did not see any changes in tobacco product usage. However, in the case of dual users, there were cases of considering or attempting to stop smoking during the pandemic. The reason for trying to quit smoking was that the odor caused by smoking soaked into the face mask. Smokers showed a negative attitude toward the smell of cigarettes on the mask and expressed it by saying that they disliked it or found it to be nasty and disgusting, necessitating frequent mask replacement. Nevertheless, the reasons for continuing cigarette use included the feeling of getting a throat hit, taste, satisfaction, or the inconvenience of HTP and e-cigarette use.

*“After smoking, the face mask also smells like a cigarette.” (F 19, cigarette user)*

*“In my case, even though I tend to wash my mouth after smoking, I wear a mask, so it seems like the smell of cigarettes stays inside the mask. I have been thinking about switching to a cigarette with a milder odor.” (F 29, cigarette user)*

### Changes in smoking perception

Some participants said they would switch from cigarettes to HTPs or e-cigarettes, but most tobacco users said that they would continue to use the current tobacco products. They planned to quit smoking for their health in the future, but the COVID-19 pandemic did not affect their current attempts to quit smoking. However, there was a general attitude of being careful about health. Non-smokers tended to reinforce their thoughts of not smoking in the future due to COVID-19. This change in attitude reinforcement indicates that non-smokers were more concerned about their health or that they had more sensitive attitudes than smokers. Smokers mostly agreed that they were at high risk for COVID-19; non-smokers agreed or did not care.

*“I have not thought about it at all, so I do not think it has any relation.” (M 30, cigarette user)*

*“Maybe because of COVID-19 stress, it is annoying when I smell it, and because of that, I thought I should stay away from cigarettes.” (M 19, non-smoker)*

### Changes in subjective mental health

Changes in daily life were expressed as more stressful for non-smokers than for smokers. Non-smokers responded that they relieved daily stress by drinking, traveling, eating, and meeting friends. However, the pandemic had limited the ways in which people relieved stress. Smokers cited reasons for smoking as, stress relief, habit, mental and physical stability, and maintaining interpersonal relationships. They responded that COVID-19 made them change their smoking place and amount of smoking. However, they did not think that this change affected their stress, explained that this was because they could still smoke when stressed in daily life.

*“I get more stressed. I need something for relief, but my routine comprises the company and home, so there is no way to refresh myself.” (F 44, HTP and cigarette user)*

*“There is no space to relieve stress. Those spaces disappeared because of COVID-19. So, it seems that more friends smoke.” (M 19, non-smoker)*

### Changes in exposure to secondhand smoke

Non-smokers who were exposed to secondhand smoke before COVID-19 continued to be exposed to secondhand smoke thereafter. Non-smokers continued relationships with family, friends, and co-workers, even though they believe that exposure to secondhand smoke harms their health. However, since COVID-19, non-smokers recognized that face masks block secondhand smoke and were less worried about the exposure than before. During the pandemic, they were tolerant of secondhand smoke from acquaintances and felt reassured with the mask. However, they were sensitive to secondhand smoke from others/strangers (people who smoke on the street).

*“This (face mask) is a protective film for me. I do not know if I will breathe the same or not; the filter operates well, and I am a lot more psychologically stable anyway. Since it filters out the smell first, it is just a little more psychologically comfortable than when I am not wearing it.” (M 22, non-smoker)*

## Discussion

In this study, we found that modifications in daily life patterns related to school and work influenced the changes in smoking place, amount, and frequency. Additionally, in some cases, there was a change in the main tobacco products used. Moreover, for non-smokers, exposure to secondhand smoke did not change after COVID-19, but they felt more tolerant toward smoking.

In earlier studies, smoking amount or frequency was found to increase or decrease due to COVID-19 [10–14]; however, the findings so far are inconsistent. In this study, smokers perceived no difference in their smoking behaviors because they did not try to quit smoking or could not quit successfully. COVID-19 did, however, significantly affect changes in smoking place and frequency.

Working from home and taking online classes due to COVID-19 meant not having to engage in social interactions and public scrutiny, creating environments that were conducive to smoking more freely. These results suggest that the change in environment was the most influential factor in smoking behavior modification.

Smoking is interpreted as nicotine addiction, and a compulsive behavioral habit [18]. The implication of smoking in behavioral patterns, such as the beginning or end of an activity, and the interval between these behaviors, is significant. The place of smoking could influence these behavioral changes significantly [19]. The behavior of not smoking in specific places, such as schools and hospitals, could be due to the pressure imposed by the place [19]. Thus, the expansion of non-smoking areas could affect the attempt to quit, or successfully quitting smoking.

The expansion of non-smoking areas is known to have a positive effect on smoking reduction and cessation, and secondhand smoke exposure also significantly decreases [20]. Smoking will reduce even in a non-smoking zone [21]. A significant correlation exists between the continuous decrease in smoking rates in Korea and other parts of the world, and the constant expansion of non-smoking areas [20]. However, HTPs and e-cigarettes can pose a challenge in the future, given that they are used regardless of place. Therefore, there is need to prepare measures such as expanding non-smoking areas in a way that does not lead to a shift in tobacco products.

Smokers changed their smoking place due to COVID-19, but they were able to cope with the discomfort caused by this change. This is because the pleasure, satisfaction, and stress relief derived from smoking have a greater effect [22, 23]. Smokers’ denial of the high risk of COVID-19 could be interpreted as a justification for their actions [24]. Smokers tend to justify smoking behavior, and COVID-19 seems to have solidified and reinforced these behaviors. However, the reluctance to use the smoking room or refraining from smoking in a crowded place cannot be interpreted to mean that smokers do not care about COVID-19. These changes are attributed to the awareness of the risk of infection when smoking in a confined place without a mask. Eventually, the behavioral change of avoiding dangerous places occurred, but smoking appeared to continue because the satisfaction from smoking provides greater rewards than the risk burden.

The reasons for smoking are stress relief and habit [25]. In this study, smokers cited stress relief, stability, satisfaction, and psychological stability as the reasons for smoking continuance, and the results were consistent with previous studies [22, 23, 25]. However, changes in daily life caused by COVID-19 could act as a stress factor for both smokers and non-smokers; the response to the stress of the pandemic was weaker among the former. Contrary to the results of this study, previous studies have found that smokers have higher levels of daily stress than non-smokers [26]. Although smokers maintained that they used tobacco products for stress relief, it should be noted that differences in stress levels and coping mechanisms are influenced by special changes in daily life. These changes are caused by special situations like the pandemic, and the way to relieve it depends on smoking status.

Differences were observed in smoking behaviors and health attitudes between smokers and non-smokers. Smokers were proud of their health and showed no concern about the risk of smoking. Non-smokers, however, were more sensitive to health than smokers. In terms of the health effects of smoking, the basic level of common sense and the choice to smoke, depend on an individual’s personal preference [27]. This suggests that attitudes and beliefs about health can influence the initiation and continuation of smoking.

Non-smokers who are exposed to constant secondhand smoke tended to value their relationships more than their health. Despite the constant exposure, they wanted to maintain their relationships with friends and co-workers. Thus, the biggest advantage during COVID-19 conferred on non-smokers is the use of face masks. Forced mask-wearing due to implemented policies protected non-smokers from exposure to secondhand smoke.

Attitudes toward masks showed different patterns. Smokers found wearing a mask to be disgusting due to the soaked smell of cigarettes. Therefore, the mask was frequently replaced or cigarettes were replaced with e-cigarettes. The recognition that e-cigarettes are “less bad” than cigarettes was the most important factor influencing the use of e-cigarettes. This could be because they are free from the unique smell of cigarettes [28, 29]. Negative reactions to cigarette odors were common to both smokers and non-smokers. They were satisfied with the reduced smell from switching to the e-cigarette or HTP. To prevent the use of e-cigarettes or HTP, it is important to effectively communicate the risk and danger that is still present, hidden in the reduced odor, to the public.

Finally, our research results show that COVID-19 is not reason enough to make smokers quit smoking. Rather, this study confirmed the possibility of a transition to the use of new types of tobacco. There is need to rethink publicity and education on smoking, as these are currently not equipped to persuade those at high risk of COVID-19 to quit smoking. It will be possible to change smoking behaviors, attract the interest of those who are not interested in smoking, and spread smoking de-normalization throughout society only when effective public messages and educational content are delivered [30].

## Data Availability

The data are not publicly available due to the risk of identifying the participants. But are available from the corresponding author upon reasonable request.
